# Aberrant activation of TGF-β1 induces high bone turnover *via* Rho GTPases-mediated cytoskeletal remodeling in Camurati-Engelmann disease

**DOI:** 10.3389/fendo.2022.913979

**Published:** 2022-10-17

**Authors:** Qi Chen, Yan Yao, Kun Chen, Xihui Chen, Bowen Li, Rui Li, Lidangzhi Mo, Weihong Hu, Mengjie Zhang, Zhen Wang, Yaoping Wu, Yuanming Wu, Fangfang Liu

**Affiliations:** ^1^ Department of Biochemistry and Molecular Biology, School of Basic Medicine, Air Force Medical University, Xi’an, China; ^2^ Shaanxi Provincial Key Laboratory of Clinic Genetics, Air Force Medical University, Xi’an, China; ^3^ Department of Cell Biology and Genetics, Medical College of Yan'an University, Yan’an, China; ^4^ Department of Anatomy, Histology and Embryology and K.K. Leung Brain Research Centre, School of Basic Medicine, Air Force Medical University, Xi’an, China; ^5^ Department of Orthopedics, The First Affiliated Hospital of Air Force Medical University, Xi’an, China; ^6^ Department of Neurobiology, School of Basic Medicine, Air Force Medical University, Xi’an, China

**Keywords:** Camurati-Engelmann disease, osteoclast, TGF-β1, Rho GTPases, cytoskeletal remodeling

## Abstract

In the adult skeleton, the bone remodeling process involves a dynamic coordination between osteoblasts and osteoclasts, which is disrupted in diseases with high bone turnover rates and dysregulated transforming growth factor beta 1 (TGF-β1). However, little is known about how TGF-β1 signaling mediates bone resorption. Here, we described a pedigree with a heterozygous variant in TGF-β1 (R218C) that resulted in aberrant activation of TGF-β1 through an activating mechanism that caused Camurati-Engelmann disease (CED). We showed that CED patients have high levels of active Rho GTPases and the migration-related proteins Integrin β1 and Integrin β3 in their peripheral blood. HEK293T cells transfected with a plasmid encoding this mutant expressed high levels of TGF-β1 and active Rho GTPases. Furthermore, activation of Rho by TGF-β1 increased osteoclast formation and bone resorption, with increased migration of pre-osteoclasts, as well as cytoskeletal remodeling of pre-osteoclasts and mature osteoclasts. Importantly, pharmacological inhibition of Rho GTPases effectively rescued hyperactive TGF-β1-induced osteoclastogenesis *in vitro*. Overall, we propose that Rho GTPases mediate TGF-β1-induced osteoclastogenesis and suggest that Rho-TGF-β1 crosstalk is associated with high bone turnover in CED.

## Introduction

Anomalies of the bone remodeling process are usually described in skeletal disorders, including Camurati-Engelmann disease (CED) (1). The disorder manifests with pain in early childhood, muscular weakness, and gait disturbances (2). Transforming growth factor beta 1 (TGF-β1) is involved in CED, and in healthy individuals, activated TGF-β1 stimulates bone deposition (3, 4). In CED, mutations cause inadequate activation of TGF-β1 and poor-quality bone formation (5); however, the role of TGF-β1 in CED remains unclear.

In skeletal tissues, TGF-β1 regulates the functions of osteoblasts and osteoclasts (1, 6–8). Specifically, TGF-β1 binds to cell surface receptors that activate receptor-regulated Smads (R-Smads), which are important proteins for regulating cell development and growth (9). In addition, TGF-β1 also acts through non-Smad effectors, including Rho GTPases, focal adhesion kinase (FAK) and mitogen-activated protein kinases (MAPKs) (10–13). Among them, Rho GTPases regulate the organization of the cytoskeleton (9), and once activated, they bind to protein kinases and actin-binding proteins that modulate F-actin dynamics, leading to morphological changes (9, 14). Recent studies found that the Rho GTPases and Rho-associated coiled-coil-containing protein kinases (ROCKs) were involved in cell growth, cell death and cytoskeletal reorganization in osteogenic cells. ROCK activity also triggered cartilage degradation and affected bone formation. In chondrocytes and osteoblasts, the inhibition of Rho GTPase/ROCK activity prevented cartilage degradation and promoted bone formation (15).

Osteoclasts maintain the balance of bone metabolism in the body by cooperating with osteoblasts. Excessive activity of osteoclasts leads to bone loss; thus, understanding the activities of osteoclasts is necessary for developing therapeutic strategies against osteoclast-related disorders (16). Although the relationship between Rho GTPases and bone metabolism is well-documented, the role of Rho GTPases in regulating TGF-β1-induced osteoclast production remains ambiguous.

Here, the clinical features of heterozygous patients with CED—with a mutation in the TGF-β1 propeptide—are described. We reported a CED pedigree that causes aberrant activation of TGF-β1 due to p.R218C mutation. Our study also confirmed that activated TGF-β1 promotes osteoclast fusion and maturation through Rho GTPase-mediated cytoskeletal remodeling and cell migration. We consider that Rho GTPases signaling is a possible therapeutic target for bone diseases with high bone turnover rates including CED.

## Materials and methods

### Genetics

Peripheral blood was obtained from individuals with CED and their relatives. The DNA extraction kit (QIAamp DNA Micro, Qiagen) was used to extract genomic DNA from white blood cells. Several sets of primers were designed to amplify segments of TGF-β1. The amplification was performed with PCR and the amplified products were analyzed by Sanger sequencing.

### Cell treatments

Cells were purchased from the American Type Culture Collection (ATCC, TIB-71). The Dulbecco’s modified Eagle’s medium (DMEM) was used to culture RAW264.7 cells, along with 10% fetal bovine serum (FBS), streptomycin (100 μg/ml) and penicillin (100 units/ml). Then, they were seeded in a chamber at 37°C, supplemented with 5% CO_2_. The ALK4/5/7 inhibitor SB431542 (MCE, 10 μM), ROCK inhibitor Y27632 (Selleck, 10 μM), or Rac GTPase inhibitor NSC23766 (Selleck, 50 μM) were used as treatments. Later, cells were exposed to recombinant sRANK Ligand (Peprotech, 30 ng/ml) and recombinant TGF-β1 (Peprotech, 10 ng/ml) and processed for further experiments.

### Osteoclast differentiation

RAW264.7 cells were cultured with density at 2×10^4^ cells/cm^2^. At 12 hours, the cells attached to the wall were medicated with RANKL and TGF-β1 (0, 1, 5, 10, and 20 ng/ml) for 5 days. The inhibitors were added to the culture solution for 1 hour before adding RANKL and TGF-β1. Cells were harvested for protein, RNA, cell staining and SEM. We conducted osteoclast differentiation experiments at least three times.

### TRAP staining assay

After washing with PBS, cells were fixed with 4% PFA. Then, 0.1% Triton X-100 permeabilized cells for 10 min. After washing, cells were stained with tartrate-resistant acid phosphatase (TRAP) solution (Sigma-Aldrich). After washing, cells were stained with hematoxylin to localize nuclei. TRAP positive cells (nuclei ≥ 3) were defined as differentiated osteoclasts. Bright-field images were captured using the inverted fluorescence microscope (Olympus IX73).

### Western blot analysis

The RIPA buffer (Biotime Biotechnology, cat. #P0013B, China) was used to extract proteins. The total protein content was analyzed through a bicinchoninic acid (BCA) kit (Biovision, cat. #K813-2500). For denaturation, a metal bath was used to boil samples for 10 min after mixing with 6× loading buffer (Tiangen, #RT201). The amount of 20 μg protein was moved into polyvinylidene difluoride (PVDF) membranes after separation using 10% SDS-PAGE. The blocking agent (5% skim milk) was then added. The PVDF was incubated with several primary monoclonal antibodies: rabbit anti-Smad2/3(CST, #8685, 1:1000), rabbit anti-p-Smad2/3 (CST, #8828, 1:1000), rabbit anti-JNK (CST, #9552, 1:1000), rabbit anti-p-JNK (CST, #4668, 1:1000), rabbit anti-ERK1/2 (CST, #4695, 1:1000), rabbit anti-p-ERK1/2 (CST, #4370, 1:1000), rabbit anti-p38 (CST, #9212, 1:1000), rabbit anti-p-p38 (CST, #4631, 1:1000), rabbit anti-Tartrate Resistant Acid Phosphatase (Abcam, #ab191406, 1:2000), mouse anti-Smad4 (Santa, #sc-7966, 1:1000), rabbit anti-NFATc1 (CST, #8032, 1:1000), rabbit anti-RhoA (CST, #2117, 1:1000), rabbit anti-Cdc42 (CST, #2466, 1:1000) and mouse anti-β-actin (Sigma, 1:10000). Rabbit polyclonal antibodies anti-Rac1/2/3 (CST, #2465, 1:1000) were also used. The secondary antibodies used were horseradish peroxidase-conjugated goat anti-rabbit and goat anti-mouse (Sigma, 1:8000). The chemiluminescence was developed with ECL solution (Milllipore). Experimental procedures were performed three times in triplicate, independently.

### Real time PCR

The AxypreTM Miniprep Kit (Axygen, cat. #365) extracted the RNA from RAW264.7 cells. The PrimeScript™ RT Master Mix (Takara, cat. #RR036A) reverse-transcribed the RNA. The real-time quantitative polymerase chain reaction (PCR) was performed by SYBR Premix Ex Taq™ II (Takara, #RR820A) and 7500 system (Applied Biosystems), followed by the operational steps: 95°C for 30 s, 40 cycles at 95°C for 3 s and 60°C for 30 s, then again 60°C for 30 s. The mRNA of GAPDH was the reference as standard control. The 2^−ΔΔCt^ method calculated the relative expression of target genes. The primers are described in **Supplementary Table 1**. The Primer 3 software (http://primer3.ut.ee/) designed oligonucleotides. We performed all PCR experiments three times in triplicate, independently.

### Resorption pit formation assay

The assay was performed in accordance with a typical assay procedure of Bone Resorption Assay Kit (CSR-BRA-48KIT, Cosmo Bio Co., LTD). The kit contains 48-well plates pre-coated with carbonate apatite (CaP). Prior to cell seeding, each well in the plate was coated with fluoresceinamine-labelled chondroitin sulphate (FACS) for 2 h. RAW264.7 cells (1 × 10^4^ cells per well) were used for the differentiation process with RANKL (30 ng/ml), TGF-β1 (10 ng/ml) and/or inhibitors. Plates were incubated at a humidity of 5% CO_2_ for 5 days at 37°C. On day 5, the conditioned medium (100 μl) was moved into a 96-well black plates. For each well, the bone resorption assay buffer (50 μl) was later added. A plate shaker was used for mixing. The fluorescence was measured according to the previously set parameters. The media were aspirated on day 5 to analyze pit formation. 5% sodium hypochlorite (100 μl) was added for 5 min. The wells dried at room temperature from 3 to 5 h after washing with distilled water. The pit areas were visualized under bright-field using a microscope. The Image J software analyzed the images. A microplate reader (Tecan Spark, Switzerland) was used to read the fluorescence signal produced.

### Transwell migration assay

The migratory response of RAW 264.7 cells was analyzed through transwell inserts (8 um, Costar, Corning, NY). RAW 264.7 cells were seeded in the upper chamber (1 × 10^5^ cells/well), with RANKL (30 ng/ml), TGF-β1 (10 ng/ml) and/or inhibitors. The inhibitors were used as pre-treatment for 1 h for 2 days. Methanol fixed migrated cells, then crystal violet stained cells. The transwell membrane was photographed through three independent views and the migrated cells were counted.

### FITC-phalloidin staining

After washing two times with PBS, 3.7% formaldehyde fixed cells and 0.1% Triton X-100 permeabilized cells for 10 min, respectively. Monolayers were blocked with 2% BSA for 30 min and seeded with 10 μg/ml of FITC-phalloidin (P5282, Sigma-Aldrich) at 37°C for 1 h. Then, the nucleus were labelled with DAPI (D9542, Sigma Aldrich, 1 μg/ml) for 5 min. After another washing with PBS, cells were visualized through a laser confocal microscope (Olympus, Japan). For each sample, random fields evaluating intracellular F-actin intensity and the formation of F-actin ring with a specific software.

### Electron microscopy

RAW264.7 cells on the plastic coverslip were treated as described. 1% osmium tetroxide and 2.5% glutaldehyde were used to fix mock and inhibitor-treated cells, followed by ethanol dehydration. After drying, silver paste was used to mount slides. Osteoclasts were imaged under ×1,000 and ×3,000 magnification using a Hitachi S-3400N SEM. In addition, for pre-osteoclasts, the magnifications were ×2,000 and ×7,000, respectively.

### Rho GTPase activation assay

The assay was implemented through a GTPase G-LISA activation assay kit (BK135, Cytoskeleton, Inc.). After washing with PBS, RAW264.7 or HEK293T cells were treated with a cold lysis buffer for 20 min. After centrifugation at 4°C, the samples were analyzed through a bicinchoninic acid (BCA) protein assay kit (Biovision, cat. #K813-2500). Around 25 μg of total proteins were loaded in GTPases binding wells for detecting the active form of Rho GTPases (RhoA, Rac1 and Cdc42). The active forms were immobilized, then primary antibodies for Rho GTPases and HRP-conjugated secondary antibodies were added. After PBS washing HRP detection substrates were added and a microplate reader (Tecan Spark, Switzerland) was used to measure the intensity by luminometric methods. Detection of lysates from peripheral blood mononuclear cells (PBMCs) was also performed according to the procedure described above.

### TGF-β1 mutants and cell transfection

Human cDNA was cloned from TGF-β1 in pCDNA 3.1 vectors. Plasmids constructed by Hunan FengHui Biotechnology Co., Ltd include pcDNA3.1-T2A-EGFP (control), TGF-β1-WT-pcDNA3.1-T2A-EGFP (WT) and TGF-β1 (C652T)-pcDNA3.1-T2A-EGFP (mutation). Medium or cell lysates were obtained for expression of mutant and WT TGF-β1 after transfection. The protein expression was quantified by enzyme-linked immunosorbent assay (ELISA), GLISA, western blotting and cell staining as described.

### Conditioned medium culture

Forty-eight hours after we transfected HEK293T cells with the above plasmids, the conditioned medium was collected and concentrated with a centrifugal filter device (3 kDa cut-off; Amicon Ultra-15, Millipore). The resulting medium (approximately 50-fold concentrated) was assayed for active TGFB1 levels by ELISA assays and preserved in aliquots at −80°C until use. When preparing to start the experiment, add concentrated conditioned medium was added to RAW264.7 cells cultured in 12-well and 24-well plates to induce osteoclasts for 5 days. We set up the following experimental groups: concentrate of untransfected HEK293T cells (293-U, with RANKL), concentrate transfected with NC plasmid (NC, with RANKL), wild-type plasmid (WT, with RANKL), mutant plasmid (MUT, with RANKL), blank control group (CON, RANKL only) and positive control group (PC, RANKL and TGF-β1 combined); the final concentration of TGF-β1 in the medium of the mutant group was 10ng/ml. Cells were then collected for western blot analysis and TRAP staining.

### ELISA test

TGF-β1 was properly quantified by the enzyme-linked immunosorbent assay (ELISA, Novus, USA) in serum and supernatants.

### Molecular structure

Human TGF-β1 was obtained from the Protein Data Bank (https://www.uniprot.org) code P01137. Graphics were generated using Swiss-Model (https://swissmodel.expasy.org).

### TGF-β1 conservation analysis

A phylogenetic tree was generated and evolutionary conservation analysis was done using the ConSurf server. Bayesian calculation method was used to calculate conservation score from the protein sequence. Here the ConSurf Server (vConSurf-2016) evaluated the conservation of TGF-β1 across species. The multiple sequence alignment is displayed in **Figure 8**. **Supplementary Figure 4** shows the residue variety for R218 variant in TGF-β1. Human wild-type TGF-β1 residues are indicated in blue and human TGF-β1 variant in red (17, 18).

### Statistical analysis

Data are presented as mean ± standard deviation (SD). Independent groups were compared using the two-tailed *t*-test. GraphPad Prism software (San Diego, CA) was used. A *p*-value below 0.05 was set as statistically significant.

## Results

### Clinical features and biochemical findings

**Figure 1:**
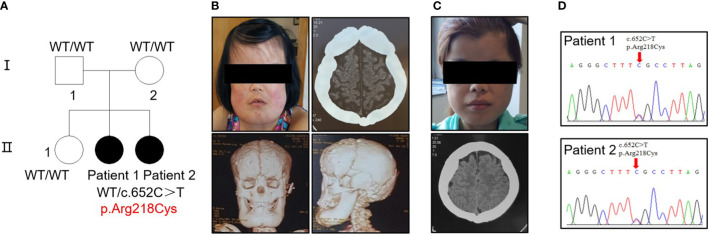
Identification of a heterozygous p.R218C mutation in patients with CED. **(A)** The pedigree covered two generations and five individuals. Patients 1 and 2 had a *de novo* mutation (p.R218C). Black-filled symbols indicate affected individuals. Patient 1 was the proband. **(B)** Clinical photograph of patient 1. A CT scan showed bilateral sclerotic thickening of the base of the skull. Images based on a reconstructed CT scan showed more pronounced symptoms. **(C)** Clinical photograph of patient 2. A CT scan showed the same symptom as patient 1. Written permission from the patients was obtained for the reproduction of these photographs. **(D)** Electropherograms of Sanger sequencing show that the TGF-β1 mutation (c.652C > T) was heterozygous in the affected individuals in this family.

Five individuals across two generations were covered by the pedigree (**Figure 1A**). The proband (patient 1, female) and her sister (patient 2, female) exhibited bone abnormalities. Computed tomography (CT) imaging showed periosteal thickening and sclerosis of facial bones and skulls (**Figures 1B, C**). Both patients suffered pain in the extremities and reported gait disturbances, protruding eyeballs and muscle weakness. They also exhibited hepatosplenomegaly, dizziness, blurred vision, and hearing loss, and patient 1 had anemia, which was absent in patient 2; however, patient 2 had changes in secondary sexual characteristics. Notably, patient 1 developed left ventricular dilation and deterioration of cardiac function, which were rarely detected in previous cases (**Supplementary Figure 1A**, **Supplementary Table 2**).

Biochemical results are displayed in **Table 1**. Serum alkaline phosphatase (ALP) and phosphorus (P) were significantly elevated in both patients (reference range: ALP, 53–141 U/L; P, 0.9–1.34 mmol/L). The thyroid hormone was in the normal range in both patients. Still, the serum thyroid-stimulating hormone (TSH) of patient 1 was significantly higher than the normal value, and her calcium level was lower than normal (reference range: 2.00–2.50 mmol/L). In addition, both patients had abnormally high activated partial thromboplastin time (APTT) and prothrombin time (PT).

**Table 1:** Overview of patients’ clinical data.

Characteristic	Patient 1	Patient 2
Gender	Female	Female
Pain in extremities	+	+
Waddling gait	+	+
Muscle weakness	+	+
Hepatosplenomegaly	+	+
Easy fatigability	+	+
Headache and dizziness	+	+
Exophthalmos	+	+
Anemia	+	–
Bone mineral density	Osteoporosis	Osteoporosis
ALP	384 U/L^1^	422 U/L^1^
P	2.14 mmol/L^1^	1.46 mmol/L^1^
Ca	1.53 mmol/L^1^	2.33 mmol/L
TG	25.31 ng/ml	12.8 ng/ml
T3	1.68 nmol/L	2.04 nmol/L
T4	99.61 nmol/L	121 nmol/L
FT3	4.51 pmol/L	4.37 pmol/L
FT4	14.86 pmol/L	17.5 pmol/L
TSH	35.09 mIU/L^1^	3.44 mIU/L
PT	13.8s^1^	14.3s^1^
APTT	43.10s^1^	52.4s^1^

^1^abnormal value. ALP, serum alkaline phosphatase; P, phosphorus; Ca, calcium; TG, thyroglobulin; T3, triiodothyronine; T4, thyroxine; FT3, free triiodothyronine; FT4, free thyroxine; TSH, thyroid-stimulating hormone; PT, prothrombin time; APTT, activated partial thromboplastin time.

Sequencing analysis identified a heterozygous transition (c.652C > T) [p.Arg218Cys] in exon 4 of TGF-β1 in the proband of each patient (**Figure 1D**). No mutation was found in their parents or siblings (**Supplementary Figure 1B**).

### A high level of active TGF-β1 in peripheral blood of CED patients is associated with bone remodeling

**Figure 2:**
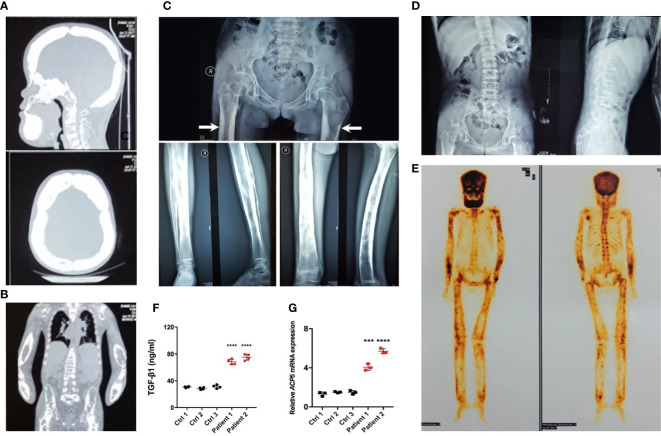
Typical osteosclerotic lesions of CED patients. **(A)** Skull radiographs of patient 1 showing bilateral sclerotic bony enlargement in the skull base and calvarium. **(B)** Thickening and irregularity of endosteal and periosteal sides of diaphyses of all long bones, including the radius and ulna. **(C)** Anteroposterior radiograph of the pelvis showing bilateral chronic femoral diaphyseal cortical thickening and sclerosis with severe degenerative changes of the hips (arrows). Lower-extremity radiographs showed bilateral symmetrical hyperostosis and endostosis of the diaphysis in both femurs, tibias, and fibulas. **(D)** Radiographs from patient 1 show severe scoliosis of the spine with osteopenia. **(E)** Whole-body bone scintigraphy of patient 1 shows the symmetrical distribution of the disease. Increased tracer uptake is visible in the diaphyseal portion of the long bones of the femora, lower legs, humeri, forearms, clavicles, and frontal bones, consistent with the radiographic findings. **(F)** The levels of active TGF-β1 were higher in peripheral blood of patients 1 and 2 than in the peripheral blood of normal controls. **(G)** The mRNA levels of *ACP5* were higher in the PBMCs of patients 1 and 2 than in normal controls. ***p < 0.001; ****p < 0.0001.

Radiographs demonstrated sclerosis of the calvarium and the skull base; bilaterally (**Figure 2A**). Imaging revealed elevated bilateral hyperostosis and endostosis of several long bone diaphysis (**Figures 2B, C**). In addition, patient 1 had severe scoliosis (**Figure 2D**). Bone scintigraphy found diffuse uptake in the skull and the upper and lower extremities, consistent with the sclerosing dysplasia lesions on radiographs (**Figure 2E**). Reduced bone densitometry values were reported for patient 1 (**Supplementary Figure 1C**). Increases in active TGF-β1 expression (**Figure 2F**) and mRNA expression of the osteoclasts marker acid phosphatase type 5 (*ACP5*) (**Figure 2G**) were described in peripheral blood and PBMCs of patients, respectively.

### TGF-β1 enhances RANKL-induced osteoclast formation

**Figure 3:**
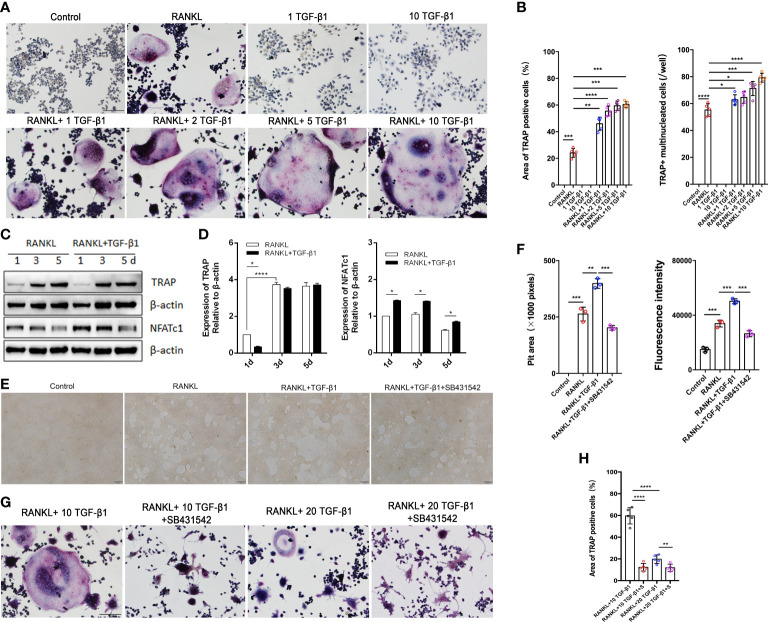
Effects of treatment of RAW264.7 cells with various concentrations of TGF-β1 for 1, 3, or 5 days in the presence or absence of RANKL. **(A)** Multinucleated cells cultured for 5 days were viewed by light microscopy and stained for TRAP. Bar: 100 μm. **(B)** The formation of multinucleated osteoclastic cells upon treatment with various concentrations of TGF-β1 was quantified by counting the number of TRAP-positive multinucleated cells and measuring the TRAP-positive cell area (n = 5). **(C, D)** Protein levels of TRAP and NFATc1 in cells cultured for 1, 3, or 5 days were determined by western blotting. **(E)** RAW264.7 cells were cultured on OsteoAssay plates for 5 days with RANKL and TGF-β1 in the presence or absence of SB431542. Resorption lacunae were visualized by bright-field microscopy. Bar: 100 μm. **(F)** Areas of resorption pits were measured. Data represent means ± SD (n = 3). **(G, H)** Cells were treated with RANKL and 10 or 20 ng/ml TGF-β1 in the presence or absence of SB431542 and then stained for TRAP. The TRAP-positive area was determined (n = 5). Bar: 100 μm. *p < 0.05; **p < 0.01; ***p < 0.001; ****p < 0.0001.

Excessive osteoclast activity leads to bone loss and osteoporosis (16). Osteoclasts were used to assess the regulatory function of TGF-β1 because of the lower bone mineral density in CED patients and the high expression of osteoclast marker ACP5 in their peripheral blood. We differentiated RAW264.7 cells in osteoclasts. The effects of different concentrations of receptor activators of nuclear factor-κB ligand (RANKL, 30 and 50 ng/ml) were first evaluated. TRAP staining showed that the effects of 30 and 50 ng/ml RANKL were similar (**Supplementary Figure 2A**); therefore, 30 ng/ml was used to induce osteoclasts in further experiments. During osteoclast formation, we administered 0, 1, 5, and 10 ng/ml of TGF-β1, which increased RANKL-induced osteoclast formation in a dose-dependent manner. Therefore, 10 ng/ml was used in the following experiments. When used alone, TGF-β1 had no osteoclast-inducing effect *in vitro*, even at a high concentration (**Figures 3A, B**).

Next, we varied the duration of exposure to these two cytokines and analyzed the osteoclast-inducing effects of TGF-β1. TGF-β1 was administered in RAW264.7 cells for different periods (**Figures 3C, D**). When RANKL was administered, exposure to TGF-β1 throughout the culture caused a time-dependent increase in the level of the osteoclast marker TRAP. Nuclear factor of activated T cells c1 (NFATc1) was significantly increased in the early stages (1–3 days). SB431542 is a potent and specific inhibitor of transforming growth factor-beta superfamily type I activin receptor-like kinase (ALK) receptors ALK4, ALK5, and ALK7. We found that treatment with SB431542 inhibited osteoclast differentiation (**Figures 3E, F**). In addition, exposure to a high concentration of TGF-β1 (20 ng/ml) reduced the number and area of multinucleated osteoclasts, and this inhibitory effect was not rescued by the addition of SB431542 (**Figures 3G, H**). These results indicate that 1–10 ng/ml of TGF-β1 could enhance RANKL-induced osteoclast formation, while a high concentration (20 ng/ml) of TGF-β1 had the opposite effect.

### The Smad and MAPK pathways regulate TGF-β1-mediated osteoclast formation

**Figure 4:**
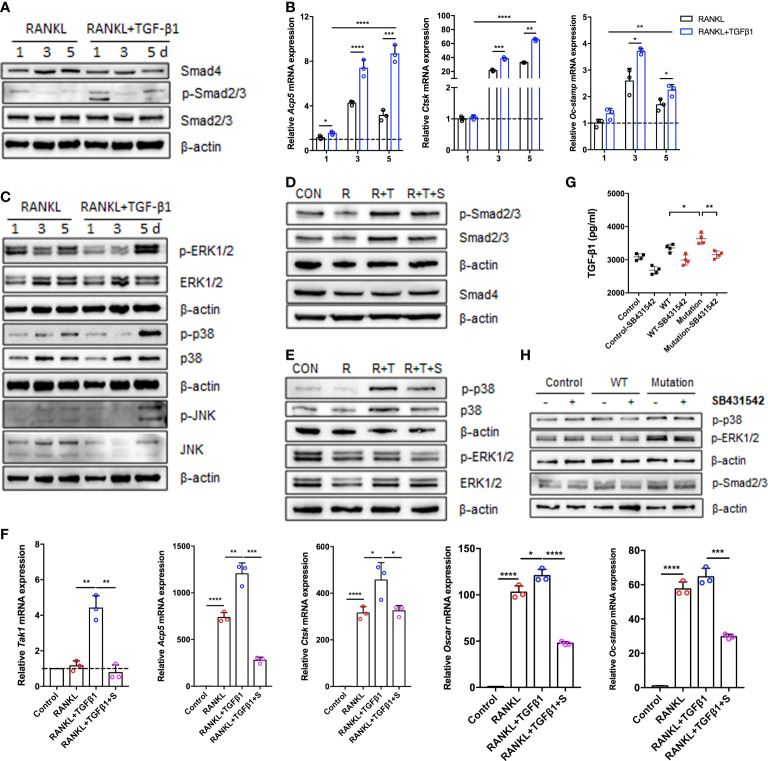
Expression of Smad and MAPK signaling pathway molecules during TGF-β1-induced osteoclastogenesis. **(A, C)** Protein levels of Smad2/3, p-Smad2/3, Smad4, ERK1/2, p-ERK1/2, p38, p-p38, JNK, and p-JNK were determined by western blotting in cells cultured with RANKL and TGF-β1 for 1, 3, or 5 days (n = 3). **(B)** Relative mRNA expression of *Acp5, Cstk*, and *Oc-stamp* was determined by qRT-PCR and normalized to that of *Gapdh* (n = 3). **(D, E)** RAW264.7 cells were cultured for 5 days with TGF-β1 and RANKL in the presence or absence of SB431542. Protein levels of Smad2/3, p-Smad2/3, Smad4, ERK1/2, p-ERK1/2, p38, and p-p38 were determined by western blotting. Representative blots are shown (n = 3). **(F)** Effects of SB431542 treatment on relative mRNA expression of *Acp5, Cstk, Oc-stamp, Osca*r, and *Tak1* were determined by qRT-PCR. These levels were normalized to that of *Gapdh* (n = 3). **(G)** HEK293T cells were transfected with the WT TGF-β1 or p.R218C mutant plasmid for 48 h. Cell supernatants were collected for an enzyme-linked immunosorbent assay (ELISA) of TGF-β1 levels (n = 4). **(H)** Western blotting of HEK293T cells transfected with the WT or mutant plasmid described above in the presence or absence of SB431542 was performed. Data represent means ± SD. *p < 0.05; **p < 0.01; ***p < 0.001; ****p < 0.0001.

To assess the effects of TGF-β1 treatment on the classical TGF-β1/Smad and non-Smad pathways during osteoclastogenesis, we analyzed the levels of mRNAs and proteins during RANKL-induced osteoclast differentiation when TGF-β1 was present. Expression of Smad4 and Smad2/3 did not significantly change, while expression of p-Smad2/3 increased in the early stage, decreased in the intermediate stage, and increased again in the late stage (**Figure 4A**). Consistent with the findings regarding osteoclastogenesis, TGF-β1 significantly increased the expression of osteoclast-associated genes induced by RANKL, including *Acp5* (also known as *Trap*), cathepsin K (*Ctsk*), and osteoclast stimulatory transmembrane protein (*Oc-stamp*) (**Figure 4B**). In addition, we investigated other effects of TGF-β1. The phosphorylation of p38, ERK, and JNK was inhibited by TGF-β1 in the early stages and stimulated in the late stages (**Figure 4C**). SB431542 inhibited phosphorylation of Smad2/3, p38, and ERK, indicating that it inhibits TGF-β1 induced Smad and MAPK activation in osteoclasts (**Figures 4D, E**). In the presence of SB431542, expression of transforming growth factor beta-activated kinase 1 (*Tak1*) was inhibited, confirming the effect of the inhibitor. The expression levels of *Acp5, Ctsk, Oc-stamp*, and osteoclast-associated receptor (*Oscar*) were significantly reduced. The enhancement of osteoclast differentiation by TGF-β1 was blocked (**Figure 4F**).

To investigate the effect of the p.R218C mutation on TGF-β1 activity *in vitro*, we constructed WT and mutant vectors investigating the effects of the p.R218C mutation on TGF-β1 activity *in vitro*. We measured the TGF-β1 protein in the supernatants of HEK293T cells transfected with these vectors using the ELISA test. TGF-β1 was higher in the supernatants of transfected cells (**Figure 4G**). Expression of the constitutively active TGF-β1 variant in HEK293T cells increased the phosphorylation of Smad2/3 and p38, but not in cells expressing WT TGF-β1 (**Figure 4H**). The TGF-β1 variant in the patients enhanced well-defined TGF-β1 receptor-mediated Smad and p38/MAPK signaling pathways and augmented osteoclast differentiation mediated by these pathways.

### Rho GTPases regulate TGF-β1-mediated pre-osteoclast migration and fusion

**Figure 5:**
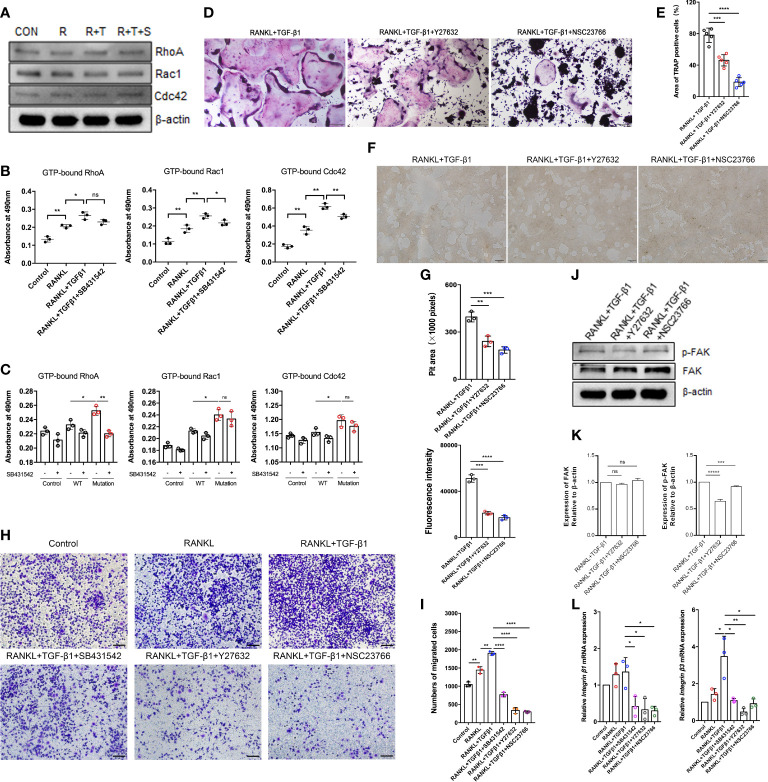
Effect of the Rho signaling pathway on TGF-β1-induced osteoclastogenesis. **(A)** RAW264.7 cells were cultured for 5 days with TGF-β1 and RANKL in the presence or absence of SB431542. Protein levels of total RhoA, Rac1, and Cdc42 were determined by western blotting. **(B)** The levels of GTP-bound RhoA, Rac1, and Cdc42 were detected by GLISAs (n = 3). **(C)** HEK293T cells were transfected with the WT TGF-β1 or p.R218C mutant plasmid for 48 h. Cell lysates were collected for GLISAs of GTP-bound RhoA, Rac1, and Cdc42 levels (n = 3). **(D, E)** Representative images of TRAP staining **(D)** (bar: 200 μm) and areas of TRAP‐positive multinucleated (≥ 3 nuclei) RAW264.7 cells **(E)** after 5 days of culture. Cells were cultured with RANKL and TGF-β1 in the presence or absence of a Rho GTPase inhibitor (Y27632 or NSC23766) (n = 5). **(F, G)** RAW264.7 cells were cultured on OsteoAssay plates for 5 days with RANKL and TGF-β1 in the presence or absence of a Rho GTPase inhibitor (Y27632 or NSC23766). Resorption lacunae were visualized by bright-field microscopy (n = 3). Bar: 100 μm. **(H, I)** Representative images of Raw264.7 cells (stained with crystal violet) on the lower surface of a Transwell membrane **(H)** and quantitative analysis of cell migration by cell counting **(I)** after 2 days of culture. Cells were cultured alone (control), with RANKL, with RANKL plus TGF-β1, or with RANKL plus TGF-β1 plus an inhibitor (SB431542, Y27632, or NSC23766) (n = 3). Bar: 100 μm. **(J, K)** Protein levels of FAK and p-FAK were determined by western blotting, and the gray values were statistically analyzed. **(L)** the mRNA levels of *Integrin β1* and *Integrin β3* were determined by quantitative RT‐PCR in RAW264.7 cells cultured for 5 days (n = 3). Data represent means ± SD. *p < 0.05; **p < 0.01; ***p < 0.001; ****p < 0.0001; *ns: no significance*.

How TGF-β1 regulates Rho GTPases to participate in osteoclast formation remains an important question in the field. To assess TGF-β1 effects on Rho signaling during osteoclast differentiation, the levels of Rho GTPases during RANKL-induced osteoclast differentiation were investigated. Expression of Rho GTPases did not significantly change overall, but the levels of GTP-bound Rho GTPases were augmented. Their levels were decreased to varying extents in the presence of SB431542 (**Figures 5A, B**), and mRNA expression of *Rock1* and *Rock2* downstream of RhoA was also reduced by SB431542 administration (**Supplementary Figure 2B**). Expression of the constitutively active TGF-β1 variant in HEK293T cells increased the levels of active Rho GTPases but not in WT TGF-β1 cells (**Figure 5C**), which was consistent with the results concerning osteoclasts.

To determine the effect of the p.R218C mutation on osteoclasts, we collected conditioned medium from HEK293T cells transfected with the plasmids to stimulate RAW264.7 cells. The results showed that the p.R218C mutant HEK293T cells-conditioned medium promoted the formation of osteoclasts differentiated from RAW264.7 cells (**Supplementary Figures 2C, D**) and activated the Smad and MAPK signaling pathways (**Supplementary Figures 2E, F**).

To further validate the role of Rho GTPases in TGF-β1-mediated osteoclastogenesis, we examined the formation of mature osteoclasts. The formation of mature osteoclasts decreased when a RhoA inhibitor (Y27632) and a Rac1 inhibitor (NSC23766) were administered. The suppressive effects of Rac1 inhibition on mature osteoclast formation (**Figures 5D, E**) and bone resorption (**Figures 5F, G**) were much greater than those of RhoA inhibition.

Spatiotemporally coordinated activation of RhoA, Rac1 and Cdc42 is required for effective cell migration. Therefore, we examined how TGF-β1 regulates osteoclast migration by activating RhoA, Rac1, and Cdc42. Transwell-based migration assays demonstrated that TGF-β1 enhanced RANKL-induced migration of pre-osteoclasts. SB431542, Y27632, and NSC23766 inhibited this migration to varying extents (**Figures 5H, I**). We determined how RhoA and Rac1 contributed to the expression of FAK and integrins involved in TGF-β1-induced osteoclast migration using Y27632 and NSC23766. Inhibition of RhoA and Rac1 downregulated RANKL- and TGF-β1-induced protein expression of p-FAK (**Figures 5J, K**) and mRNA expression of *Integrin β1* and *Integrin β3* (**Figure 5L**) according to western blotting and qRT-PCR, respectively.

To fully assess the functional consequences of the p.R218C substitution, we transfected HEK293T cells as described earlier. Transwell-based migration assay showed that the constitutively active TGF-β1 variant of HEK293T cells migrated more substantially than WT (**Supplementary Figures 2G, H**), which was consistent with the results for pre-osteoclasts. The TGF-β1 variant in the patients enhances the well-defined TGF-β1 receptor-mediated Rho signaling pathway and augments cell migration mediated by this pathway.

### Rho GTPases mediate TGF-β1-induced migration of pre-osteoclasts through cytoskeletal remodeling

**Figure 6:**
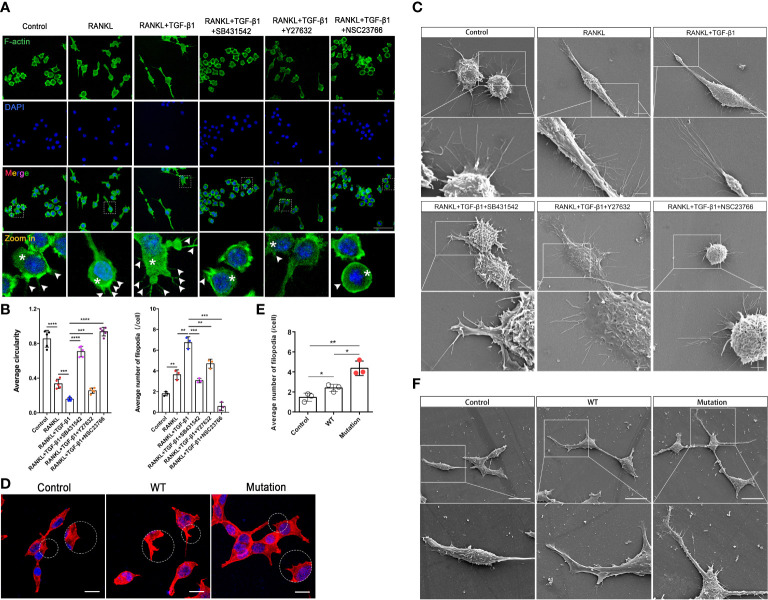
Inhibition of Rho GTPases suppresses migration of RAW264.7 cells by disrupting the actin cytoskeleton. **(A)** RAW264.7 cells were pretreated with an inhibitor (SB431542, Y27632, or NSC23766) for 24 h, treated with RANKL plus TGF-β1 for 10 min, fixed, permeabilized, and stained with phalloidin to visualize F-actin. TGF-β1-treated cells had well-defined intracellular F-actin (white asterisks) and filopodia (white arrows). Cells treated with inhibitors had less intracellular F-actin and fine aggregates of F-actin. The inhibitors caused marked morphological changes and reduced cell size, especially during treatment with NSC23766, following which cells contained little intracellular F-actin and tended to be round. **(B)** Formation of filopodia and average roundness of the different groups of cells in A (n = 3). Data represent means ± SD. **p < 0.01; ***p < 0.001; ****p < 0.0001. **(C)** Scanning electron microscopy showed that the inhibitors suppressed the formation of filopodia and membrane ruffles. Images (2000× and 7000× magnification) were captured using a Hitachi S-4800 FEG scanning electron microscope. NSC23766 treatment disrupted the actin cytoskeleton and caused shrinkage of RAW264.7 cells. Bar: 5 μm (2000×), 2 μm (7000×). **(D, E)** HEK293T cells were cultured for 16 h, transfected with different plasmids for 48 h, fixed, permeabilized, and stained with phalloidin to visualize F-actin. Cells transfected with the mutant plasmid had well-defined intracellular F-actin and more filopodia. Bar: 50 μm. **(F)** HEK293T cells were cultured for 16 h and transfected with different plasmids for 48 h, and then pseudopod expression was analyzed using scanning electron microscopy. Bar: 20 μm (1500×).

Actin dynamics are inextricably linked to osteoclast differentiation (19, 20). We hypothesized that Rho GTPases regulate pre-osteoclasts migration through the cytoskeleton reorganization. TGF-β1 significantly promoted the formation of intracellular F-actin and filopodia in RAW264.7 cells, and SB431542, Y27632, and NSC23766 inhibited this effect. Treatment with these inhibitors, especially the Rac1 inhibitor, increased the roundness of RAW264.7 cells. These cells had fewer filopodia and lamellipodia, which is likely not conducive to migration and fusion of pre-osteoclasts (**Figures 6A, B**). These findings were validated in RAW264.7 cells by scanning electron microscopy (**Figure 6C**). Then, using the previously described plasmids, we expressed the constitutively active TGF-β1 variant that promoted actin recombination in HEK293T cells, as revealed by pre-osteoclasts, but to a lesser extent in WT TGF-β1 (**Figures 6D–F**. Complete information is in **Supplementary Figure 3A**). Rho signaling mediated TGF-β1-induced pre-osteoclast migration through cytoskeletal remodeling, with Rac1, in particular, playing a more important role.

### Rho GTPases are associated with cytoskeletal remodelling during TGF-β1-induced osteoclastogenesis

**Figure 7:**
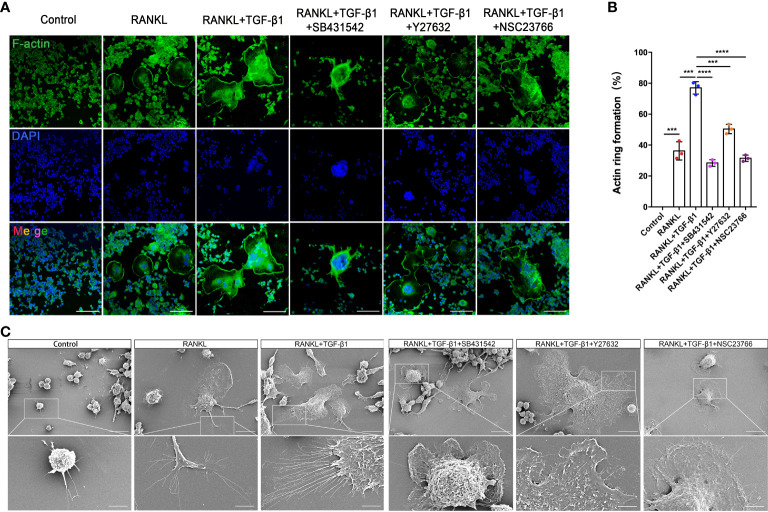
Inhibition of Rho GTPases suppresses the formation of osteoclasts from RAW264.7 cells by disrupting the actin cytoskeleton. **(A)** Actin (green) and nuclei (blue) were stained in osteoclasts cultured with RANKL, RANKL plus TGF-β1, or RANKL plus TGF-β1 plus an inhibitor (SB431542, Y27632, or NSC23766). Bar: 100 μm. **(B)** The percentage of osteoclasts with closed actin rings at the cell periphery (n = 3). Data represent means ± SD. ***p < 0.001; ****p < 0.0001. **(C)** Scanning electron microscopy demonstrated that the inhibitors suppressed the formation of filopodia and membrane ruffles. Images (1000× and 3000× magnification) were captured using a Hitachi S-4800 FEG scanning electron microscope. Treatment with SB431542 or NSC23766 disrupted the actin cytoskeleton and inhibited the formation of larger osteoclasts from RAW264.7 cells. Bar: 20 μm (1000×), 5 μm (3000×).

RAW264.7 cells showed increased cell-cell fusion and formed large multinucleated TRAP-positive osteoclasts during RANKL-induced differentiation. RANKL activation produced an actin ring in close proximity to osteoclasts. In addition, TGF-β1 promoted the formation of a larger actin ring and more abundant intracellular F-actin. Treatment with SB431542 led to osteoclasts forming smaller and contracted actin rings, while cells treated with Y27632 or NSC23766 lacked abundant F-actin in the cytoplasm, despite having larger actin rings (**Figures 7A, B**). Next, the formation of membrane folds and changes to filopodia and pseudopods were confirmed by scanning electron microscopy. The activation of TGF-β1 led to the formation of membrane ruffles and filopodia in mature osteoclasts—both actin-based structures are required for cell migration. Treatment with the aforementioned inhibitors, especially the Rac1 inhibitor, significantly reduced the formation of these structures (**Figure 7C**). TRAP staining of osteoclasts treated with these inhibitors yielded consistent results (**Supplementary Figure 3B**).

### The TGF-β1 variant demonstrates a gain of function

**Figure 8:**
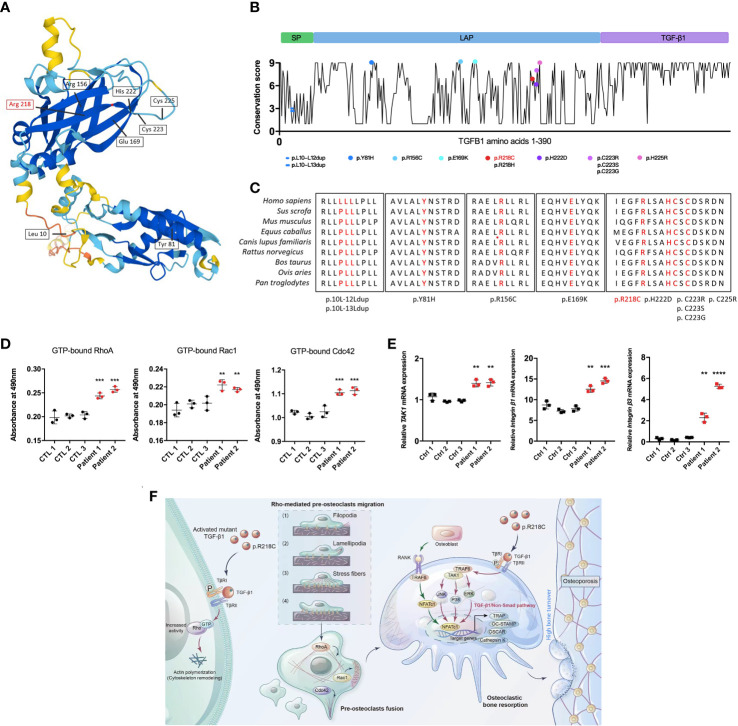
TGF-β1 variants in CED demonstrate a gain of function. **(A)** Structural model of WT human TGF-β1, highlighting the sites of TGF-β1 gain-of-function variants. **(B)** A graphical illustration of TGF-β1 sequence conservation (black line) based on the ConSurf conservation score (see Methods). The TGF-β1 domain structure and positions of the variants identified in patients are indicated, including those reported in this study (red symbols). **(C)** Multiple species alignment of TGF-β1 protein sequences at all the mutant amino acid positions reported in CED patients. Amino acids p.L10, p.L11, p.L12, p.L13, p.Y81, p.R156, p.E169, p.R218, p.H222, p.C223, and p.C225 of TGF-β1 all show species conservation. Red indicates the mutation site in the patients in this study. The red label is the variant we reported. **(D)** Levels of GTP-bound RhoA, Rac1, and Cdc42 in PBMCs from patients 1 and 2 were determined by GLISAs. **(E)** mRNA expression of *Integrin β1*, *Integrin β3*, and *TAK1* in PBMCs from patients 1 and 2 was determined by qRT-PCR (n = 3). Data represent means ± SD. **p < 0.01; ***p < 0.001; ****p < 0.0001. **(F)** TGF-β1 variants in CED patients lead to increased Rho GTPase activity through aberrant activation, resulting in Rho GTPase-mediated remodeling of the cytoskeleton: (1) sensing of the motogenic signal by filopodia, (2) formation and protrusion of lamellipodia and pseudopodia-like forward extension, (3) attachment of protrusions to the extracellular matrix, (4) F-actin-mediated contraction of the cell body to allow forward progress, and rear release. It promotes pre-osteoclast migration, fusion, osteoclast formation, and bone resorption, ultimately leading to osteoporosis.

Twelve TGF-β1 mutations that cause CED have been reported based on OMIM (https://omim.org/) data. These mutations are divided into three main categories according to the effect on the protein, namely, mutations located in exon 1, including leucine insertion in the signal peptide region (p. L10_L12dup and p.L10_L13dup) and a missense mutation at the N-terminus of LAP (p.Y81H) (21); mutations located in exon 2 including two missense mutations at the N-terminus of LAP (p.R156C and p.E169K) (22); and mutations located in exon 4, i.e., C-terminal missense mutations of LAP (p.R218C, p.R218H, p.H222D, p.C223R, p.C223S, p.C223G, and p.C225R) (23) (**Figures 8A, B**). All pathogenic mutations eventually increase the proportion of TGF-β1 in the circulation and bone, causing bone hyperplasia, accelerated bone turnover, inhibition of normal bone mineralization and decreased bone density. The mutation (p.R218C) reported here accounts for more than 60% of all mutations and is considered a hotspot mutation for CED (24). Given that TGF-β1 mutants are involved in the initiation of CED, we assessed the evolutionary conservation of the mutant acid interface. From the ConSurf server results, it was found that, all the reported pathogenic TGF-β1 variants were located in highly conserved regions, signifying that they have a critical role (**Figure 8C**). For R218, none of the missense amino acids were reported in the TGF-β1 homologs (**Supplementary Figure 4**).

To determine the functional consequences of the TGF-β1 mutation in patients regarding Rho GTPases, we examined their activation. The GTP-bound Rho GTPases were higher in PBMCs from CED patients than in healthy adults (**Figure 8D**). Likewise, the mRNA of *TAK1, Integrin β1*, and *Integrin β2* were augmented in the peripheral blood of the CED patients (**Figure 8E**). These data indicated that the TGF-β1 R218C variant induces actin remodeling by activating downstream signals. This variant led to constitutive Rho GTPases activation, consistent with the effects in osteoclasts and HEK293T cells.

We propose that in pathological conditions resulting from a gain-of-function mutation of TGF-β1 [p.Arg218Cys], well-defined TGF-β1 receptor-mediated Rho signaling in precursor cells is hyperactivated, leading to cytoskeletal remodeling. These actin-based activities lead to increased migration of precursor cells that more readily aggregate and fuse into large osteoclasts to perform their functions, leading to excessive bone resorption and disturbances in bone coupling (**Figure 8F**).

## Discussion

In this study, we described two patients with CED, usually characterized by pain at the extremities, gait disturbances, fatigue, muscle weakness, headaches, ophthalmopathy, and otological symptoms (25, 26). Our patients had these symptoms, although the incidence of hearing disturbances in CED is only 18% (5). Mutations of TGF-β1 are the genetic cause of CED (27); thus the diagnosis was performed by genetic testing of TGF-β1 in our patients. Both patients were heterozygous for c.652C > T [p.Arg218Cys] in the TGF-β1 gene. Furthermore, patient 1 exhibited left ventricular dilatation and decreased cardiac function, which were rarely reported previously. The TGF-β1 gene polymorphisms might predispose individuals to heart conditions, and several cardiomyopathies are associated with elevated TGF-β1 (28, 29). The aforementioned reports led to speculation that the predisposition might be related to aberrant activation of TGF-β1 caused by the TGF-β1 mutation in CED patients.

Twelve TGF-β1 mutations that cause CED have been identified based on OMIM (https://omim.org/), which might lead to an increased expression of pro-inflammatory cytokines (30). Studies of the influences of TGF-β1 on osteoblasts and osteoclasts have yielded contradictory effects (31); however, our study suggests that TGF-β1 maintains a biphasic effect on the differentiation of osteoclasts. Specifically, osteoclast differentiation is enhanced by low TGF-β1 and inhibited by high TGF-β1, consistent with a previous report (32). This highlights the influences of TGF-β1 on bone biology. More importantly, the latent TGF-β1 level was 68–75 ng/ml in peripheral blood cells, which was more than two times higher than that in normal controls (~30 ng/ml). In a previous study, PBMCs from CED patients contributed to the formation of more osteoclasts than healthy controls in the presence of a range of RANKL concentrations (2). We described that treatment with 10 ng/ml TGF-β1 increased the formation of osteoclasts from RAW264.7 cells substantially, but RAW264.7 cells did not form osteoclasts in the absence of RANKL, indicating that TGF-β1 cannot replace RANKL for the stimulation of osteoclast differentiation and function.

It has been reported that HEK293T cells transfected with p.R218C exhibit activated Smad signaling and secrete an increased amount of active TGF-β1 into the culture medium (21). Our observations complement the results of this previous study. We found that not only TGF-β1/Smad signaling, but also TGF-β1/non-Smad signaling, was activated in HEK293T cells transfected with the mutant plasmid and osteoclasts induced with exogenous TGF-β1, including MAPK and Rho GTPase signaling. Rho GTPases are regulators of cytoskeletal organization (33). Here, we reported that active Rho GTPases are higher in the peripheral blood of CED patients than in that of healthy controls. Consistent results were obtained with transfected HEK293T cells with a mutant plasmid. Furthermore, the levels of active Rho GTPases and mRNA expression of *Rock1* and *Rock2* were augmented in osteoclasts treated with TGF-β1. The TGF-β1 R218C mutation in CED patients presumably activates the Rho signaling pathway.

Small GTPases and ROCKs participate in cytoskeletal reorganization and differentiation toward osteogenic cells (15). These findings support the idea that RhoA/ROCK are indispensable for skeletal metabolism. We confirmed that the TGF-β1 receptor inhibitor SB431542 inhibited the activation of Rho GTPases in RAW264.7 cell-induced osteoclasts and HEK293T cells, while Rho GTPase inhibitors, including Y27632 and NSC23766, significantly reduced bone effects induced by TGF-β1. In addition, the mRNA levels of the cell migration-related molecules *Integrin β1* and *Integrin β3* were increased in the peripheral blood of CED patients. Our study further confirmed the promoting role of Rho signaling in TGF-β1-mediated osteoclastogenesis.

Excessive bone degradation by osteoclasts leads to characteristic diseases such as CED; however, how Rho GTPases are regulated by TGF-β1 in osteoclast formation remains elusive. Osteoclasts use podosomes, structures composed of F-actin and integrins, to attach to bone surfaces. The binding of integrins to ligands promote osteoclast adhesion, migration and bone resorption (34, 35). We found that TGF-β1 promoted cytoskeletal remodeling in precursor and mature osteoclasts, as manifested by an increased number of filopodia and abundant intracellular F-actin, and SB431542, Y27632, and NSC23766 partially inhibit this phenomenon. Active osteoclasts form a stable actin ring (36), and our results confirmed that TGF-β1 contributes to its formation. However, results with Rho inhibitors indicated an abolishment of the proper actin cytoskeletal network.

Meanwhile, increased migration of precursor cells, elevated expression of osteoclast-related molecules (ACP5, OSCAR, TRAP, OC-STAMP, and Ctsk), and increased bone resorption pits revealed the increased formation of mature osteoclasts. HEK293T cells transfected with the TGF-β1 mutant plasmid also exhibited changes to their cytoskeleton and migration. Therefore, we propose that the pathogenic mechanism underlying CED involves the acceleration of osteoclastogenesis and bone resorption by TGF-β1 through Rho GTPase-mediated cell migration and cytoskeletal remodeling, resulting in accelerated bone turnover, decreased bone density and osteoporosis.

There is no definitive treatment for CED. The most common treatments are corticosteroids, nonsteroidal anti-inflammatory drugs (NSAIDs) and bisphosphonates to reduce limb pain. Corticosteroids may counteract the increased activity of TGF-β1 and relieve some symptoms (37); however, they can also cause a reduction in bone density and inhibition of TGF-β1-induced transcription (38). The administration of NSAIDs does not usually elicit an efficient response (39), and the use of bisphosphonates is also disputed (37, 40). Losartan has recently been used to treat patients with CED; however, there were some limitations in terms of safety and efficacy (5, 38). In addition to CED, enhanced TGF-β1 has been documented in several connective tissue disorders (8, 41–43). It might be crucial to attenuate the effects of TGF-β1 signaling for these disorders.

In the future, a treatment approach that modulates the TGF-β1 signaling pathway might be effective in patients with CED (5). In a recent study, the inhibition of RhoA/ROCK prevented aging-associated bone loss (44). Similarly, our findings raise the possibility of a treatment for diseases in which TGF-β1 is abnormally activated, including CED. Moreover, the inhibition of Rho GTPases rescues the imbalance in bone coupling and excessive bone resorption.

In summary, we provided new clues for the pathogenesis of CED, highlighting the role of Rho GTPases in osteoclast differentiation and function and proposing their potential as targets for treating osteoporosis pathology. It is important to explore how the activation of TGF-β1/Rho GTPases leads to more specific manifestations of CED and whether inhibition of Rho GTPases improves therapeutic effects using animal models. Our new understanding of how the TGF-β1 mutation-triggered overactivation of Rho GTPases in CED leads to high bone turnover will help address the specific mechanisms of decreased bone density or osteoporosis due to aberrant TGF-β1 signaling in related diseases and inform the development of new therapeutic approaches.

## Data availability statement

The original contributions presented in the study are publicly available. This data can be found here: GenBank, 2631956.

## Ethics statement

The studies involving human participants were reviewed and approved by The Ethics Committee of Air Force Medical University. The patients/participants provided their written informed consent to participate in this study. Written informed consent was obtained from the individual(s) for the publication of any potentially identifiable images or data included in this article.

## Author contributions

YuW initiated the project and discovered the genetic variant. QC contributed to the idealization of the project, experimental design, data curation and collection, drafting the manuscript and statistical analysis. YY contributed to cell experiments and data analysis, KC contributed to the idealization and administration of the project and editing of the manuscript. XC contributed to clinical data and editing of the manuscript. BL and RL contributed to the methodology, collection of data and structural analysis. LM, WH, MZ, and ZW contributed to manuscript writing. YW, YuW, and FL contributed to the research concept, idealization of the project and drafting the manuscript. All authors approved the current version of the study.

## Funding

This study was supported by grant/award in Shaanxi (Grant/Award Number: 2019SF-059 and 2020SF-204), the Key Innovative Project in Shaanxi (Grant/Award Number: 2021ZDLSF02-02) and the National Natural Science Foundation of China (Grant/Award Number: 81671476 and 31570906).

## Acknowledgments

We thank all study participants and their family for their contribution to the study. I (FL) want to thank my newborn son (Chufan Chen) for his great support.

## Conflict of interest

The authors declare that the research was conducted in the absence of any commercial or financial relationships that could be construed as a potential conflict of interest.

## Publisher’s note

All claims expressed in this article are solely those of the authors and do not necessarily represent those of their affiliated organizations, or those of the publisher, the editors and the reviewers. Any product that may be evaluated in this article, or claim that may be made by its manufacturer, is not guaranteed or endorsed by the publisher.
