# Supplementary material for: Aberrant activation of TGF-β1 induces high bone turnover via Rho GTPases-mediated cytoskeletal remodeling in Camurati-Engelmann disease
Source: Front Endocrinol (Lausanne). 2022 Oct 17;13:913979. doi: 10.3389/fendo.2022.913979 (PMC9621586; doi:10.3389/fendo.2022.913979)
Supplement: Supplementary file 1 [file DataSheet_1.docx]

Supplementary Material

# Supplementary Figures and Tables

## Supplementary Figures


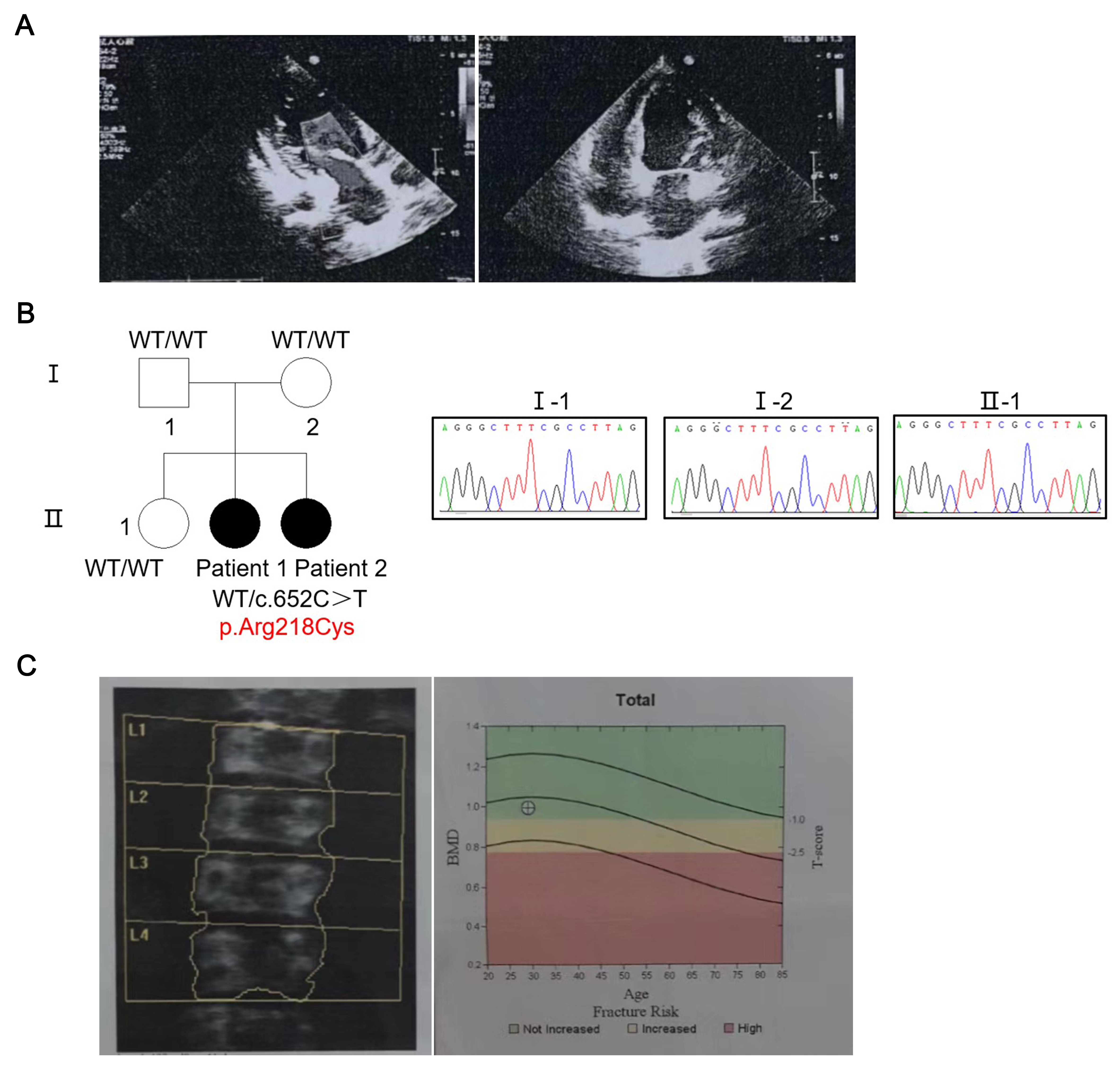


**Supplementary Figure 1.** Sanger sequencing results for other members of the family and supplementary clinical data of Patient 1. **(A)** Cardiac color ultrasound image of patient 1. **(B)** Sanger sequencing results for other unaffected members of the family. **(C)** bone densitometry of the lumbar region was performed in patient 1.


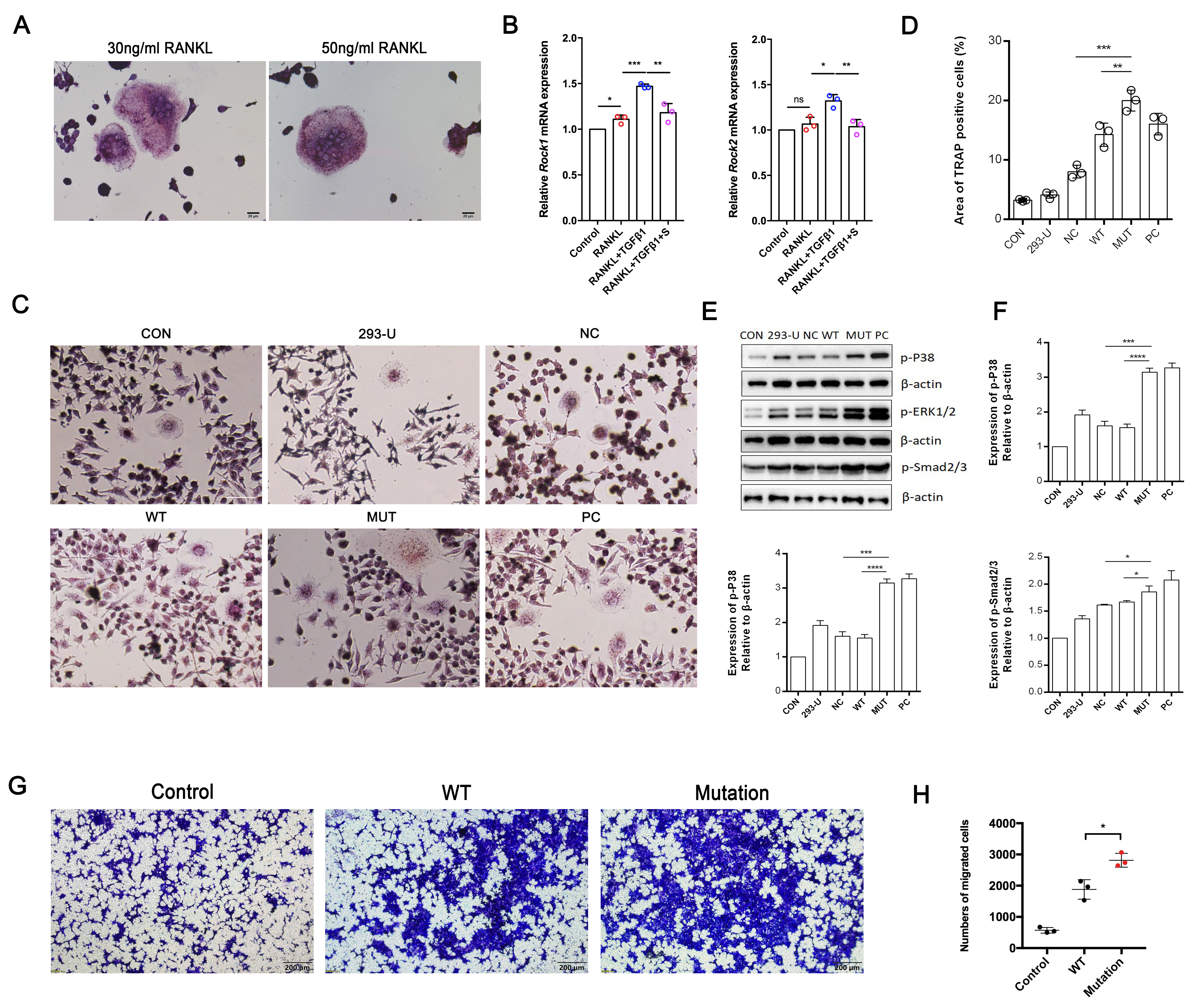


**Supplementary Figure 2.** Effects of different concentrations of RANKL on osteoclast differentiation and TGF-β1 mutation on HEK293T cell migration and osteoclast formation. **(A)** TRAP staining results of osteoclasts formation induced by different concentrations of RANKL (30 and 50 ng/ml). Bar: 20 μm. **(B)** mRNA expression of *Rock1* and *Rock2* downstream of RhoA in the presence of SB341542 (n=3). **(C, D)** TRAP staining and statistical results of osteoclast formation induced by conditioned medium of p.R218C mutant HEK293T cells. Bar: 50 μm. **(E, F)** The effects of TGF-β1 mutation on Smad and MAPK signaling pathways during osteoclasts differentiation were assayed by conditioned medium. CON: blank control group (RANKL only); 293-U: concentrate of untransfected HEK293T cells (with RANKL); NC: concentrate transfected with NC plasmid (with RANKL); WT: concentrate transfected with wild-type plasmid (with RANKL); MUT: concentrate transfected with mutant plasmid (with RANKL). **(G, H)** Transwell assay-based migration ability analyses of HEK293T cells. The cells were transfected with indicated plasmids for 48 h prior to analysis (n=3). Bar: 200 μm. Data represent means ± SD. **p < 0.05; **p < 0.01; ***p < 0.001; ****p < 0.0001; ns: no significance.*


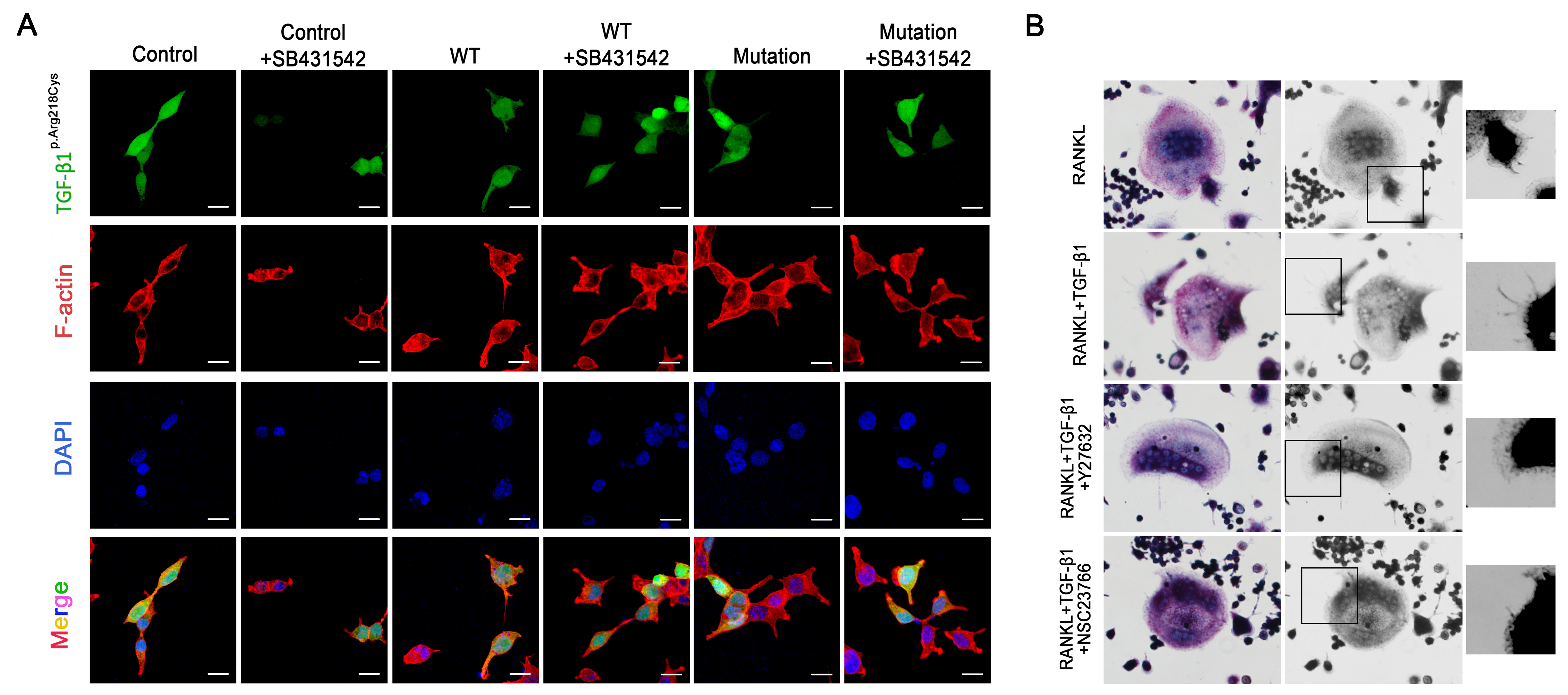


**Supplementary Figure 3.** Inhibitors inhibit the growth of TGF-β1-induced F-actin. **(A)** Staining analysis of intracellular F-actin and filopodia in HEK293T cells. The cells were transfected with the indicated plasmids for 48 h and treated with SB431542 for 24 h prior to analysis. Bar: 50 μm. **(B)** TRAP staining results of inhibitor-treated osteoclasts.


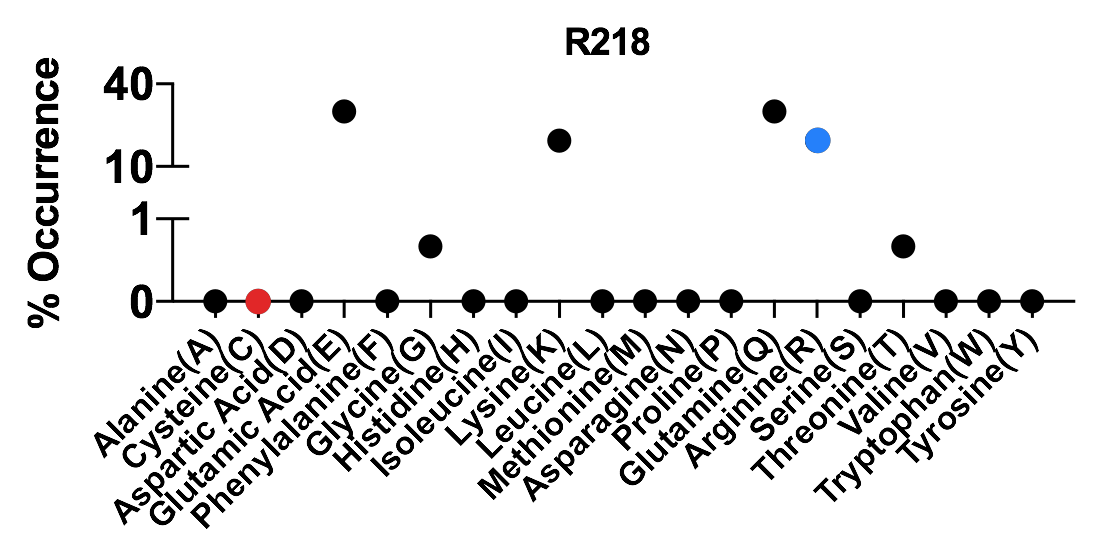


**Supplementary Figure 4.** Residue variety in % for p.R218C. Summary of the residue variety in % for p.R218C variant in TGF-β1 following ClustalW-based multiple sequence alignment using the ConSurf server (see Methods). Human wild-type TGF-β1 residues are indicated in blue and human TGF-β1 variant in red.

## Supplementary Tables

**Supplementary Table 1.** List of oligonucleotides used for qRT-PCR analyses.

| **Species** | **Gene** | **Forward Primer (5’-3’)** | **Reverse Primer (5’-3’)** |
| --- | --- | --- | --- |
| Mouse | *Oscar* | TGCAGTATATTGACTCTGTGC | CAGAACCTTCGAAACTGATGAC |
|  | *Tak1* | TCCGAGGGCAAGAGGATGAGT | CCAGGTTCTGTCCCAGTAACAGTC |
|  | *Oc-stamp* | CTGCGTTTCGACAATATCTACG | TACTAAGAGCAATGCCAGTAGC |
|  | *Acp5* | CAAGAACTTGCGACCATTGTTA | ATCCATAGTGAAACCGCAAGTA |
|  | *Ctsk* | TGGACTGTGTGACTGAGAATTATGG | CCGTTCTGCTGCACGTATTG |
|  | *Integrin β1* | TCACATGCAGGTTTGGAAAA | TCACAATGGCACACAGGTTT |
|  | *Integrin β3* | TGCTCCAGAGTCTATTGAGTTCC | GAGAAAGACAGGTCCATCAAGTAG |
|  | *Rock1* | TTCATTCCTACCCTCTACCACTTTC | TTAACATGGCATCTTCGACACTCT |
|  | *Rock2* | TTGGTTCGTCATAAGGCATCAC | TGTTGGCAAAGGCCATAATATCT |
|  | *Gapdh* | AGGTCGGTGTGAACGGATTTG | TGTAGACCATGTAGTTGAGGTCA |
| Human | *ACP5* | CTTTGTAGCCGTGGGTGACT | GGGAGCGGTCAGAGAATACG |
|  | *TAK1* | CAAAGCTAAGTGGAGAGCAAAAGA | GATAACTGCCGAAGCTCTACAATAA |
|  | *INTEGRIN β1* | TTCGATGCCATCATGCAAGTTG | CCATCTCCAGCAAAGTGAAACC |
|  | *INTEGRIN β3* | CATCCTGGTGGTCCTGCTCT | GCCTCTTTATACAGTGGGTTGTT |
|  | *GAPDH* | GCACCGTCAAGGCTGAGAAC | TGGTGAAGACGCCAGTGGA |

**Supplementary Table 2.** Results of cardiac ultrasonography.

| **Parameters** | **Patient 1** | **Reference range** |
| --- | --- | --- |
| Aorta ascendens (Asc Ao) | 3.4cm | 2.1-3.4cm |
| Aortic sinus (Sinus) | 3.2cm | 2.1-3.5cm |
| Left atrium (LA) | 3.6cm | 1.9-4.0cm |
| Right ventricle (RV) | 1.6cm |  |
| Interventricular septum (IVS) | 0.9cm | 0.6-1.1cm |
| Left ventricular end diastolic dimension (LVDd) | 5.7cm^1^ | 3.5-5.5cm |
| Left ventricular posterior wall (LVPW) | 0.8cm | 0.6-1.1cm |
| Pulmonary artery (PA) | 2.2cm | 1.2-2.6cm |
| Right ventricular outflow tract (RVOT) | 2.8cm |  |
| Left ventricular end-diastolic volume (EDV) | 159ml |  |
| Left ventricular end-systolic volume (ESV) | 94ml |  |
| Ejection fraction (EF) | 40%^1^ | 50-75% |
| Fractional shortening (FS) | 20% |  |
| Color Doppler flow imaging showed moderate mitral regurgitation, and revealed that the  E peak＜A peak of the mitral valve blood flow during diastolic filling. ^1^ | | |

^1^ abnormal value.
